# Sticking with the old seed: Input value chains and the challenges to deliver genetic gains to smallholder maize farmers

**DOI:** 10.1177/0030727019900520

**Published:** 2020-01-20

**Authors:** Pieter Rutsaert, Jason Donovan

**Affiliations:** 1 International Maize and Wheat Improvement Centre (CIMMYT), Nairobi, Kenya; 2 International Maize and Wheat Improvement Centre (CIMMYT), Texcoco, Mexico

**Keywords:** Seed systems, agro-dealers, hybrid maize, push–pull marketing, Kenya

## Abstract

The Kenyan maize seed sector exhibits high hybrid adoption rates, a growing number of seed companies, and an extensive agro-dealer network. Nonetheless, maize yields remain low and uptake of new, stress-tolerant varieties has been disappointing. This article investigates interactions in maize seed value chains in Kenya, and decisions made by agro-dealers, farmers, and seed companies, to gain a better understanding of how to encourage the uptake of new, stress-tolerant varieties. Data were collected during the 2019 seed-purchasing season from Kenyan seed companies (*n* = 8), agro-dealers (*n* = 80), and farmers immediately following their seed purchase (*n* = 466). Most agro-dealers had a wide offer of seed products available, but seed companies’ engagement with them was limited and marketing efforts were directly focused on farmers. Only a fraction of farmers used the agro-dealer environment to guide their decision-making. However, when agro-dealers engaged with farmers, they influenced varietal selection in 80% of the cases. Agro-dealers were one of the key information outlets about maize seed varieties. Seed company engagement with agro-dealers and in-store promotions (push marketing) should be further explored to improve returns on investments in seed systems.

## Introduction

Crop genetic improvement has been crucial in feeding the world population (Lipton and Longhurst, 1989) and has resulted in 20–50% yield growth in developing countries between 1960 and 2000 (Evenson and Gollin, 2003). The high social return of crop genetic improvement has led to substantial investments in agricultural research and development (AGRA, 2017; Toenniessen et al., 2008). While landraces have been replaced by modern varieties for several crops, the slow speed of varietal turnover has become a topic of concern and discussion (Spielman and Smale, 2017; Walker and Alwang, 2015). Climate change is predicted to negatively impact yields in food-insecure regions in Africa through higher average temperatures (Cairns et al., 2013), more frequent extreme weather events (Lesk et al., 2016), as well as increased pest and disease prevalence (Chakraborty et al., 2000), thus underscoring the need for future investments in crop breeding. Over the past decade, in addition to higher yield potential, the Consultative Group for International Agricultural Research (CGIAR) breeding programs have focused on stress tolerance, including drought tolerance, pest tolerance, and low soil fertility which is critical to mitigate these climate change risks (Atlin et al., 2017). However, adoption of these new, stress-resilient varieties continues to lag, even in countries like Kenya, where overall hybrid maize adoption rates are high.

Debates on formal seed systems have addressed both the supply and demand constraints faced by actors along the seed value chain. On the supply side, researchers have pointed to various issues that limit the capacity of seed companies to produce increasing volumes of quality seed, including lengthy varietal release process, limited access to early generation seed, and a policy environment which limits seed business growth and development (Erenstein and Kassie, 2018; Langyintuo et al., 2010; Spielman and Smale, 2017). On the demand side, the literature has focused mainly on the factors which predict seed adoption by farmers (Abate et al., 2017; Fisher and Carr, 2015; Simtowe et al., 2019). However, in neither of these discussions has the role of input value chains in driving or hampering hybrid seed sales been adequately addressed. While food value chains are quite well researched, this is not the case for the competitive environment of the input sector in agriculture (Sheldon, 2016). This knowledge gap persists despite the large investments being made to expand formal seed systems in sub-Saharan Africa to make agricultural inputs more accessible.

The agro-dealer space is where farmers, especially those in remote rural areas, obtain access to various inputs for maize production, including seed, and related information on input performance across different contexts (Access to Seeds Foundation, 2019). Unlike seed distribution by development projects and government agencies, agro-dealers are more likely to maintain their presence in a given place over time. Researchers have advocated the potentially important role of agro-dealers in supporting farmers’ decisions on input purchases (Allgood, 2011) and have described the constraints they face to expand and consolidate their businesses (Adesina et al., 2014; Odame and Muange, 2011). However, these discussions to date have tended to be descriptive in nature and the role of agro-dealers in varietal turnover has been neglected. Also, from a farmer perspective, the seed selection process and the role of the agro-dealer in shaping farmers’ decisions have not been investigated to the best of our knowledge.

The agro-dealer space offers a strategic entry point for interventions to accelerate varietal turnover in the maize seed sector. However, a better understanding is needed of the underlying factors that influence interactions between seed companies, seed retailers, and farmers. This study explores the choices and strategies of agro-dealers regarding maize seed sales as well as insights into how they engage with seed suppliers as well as customers; secondly, the purchase decisions farmers make at the agro-dealer, and third, the view of seed companies on their agro-dealer networks. The following four questions guide our analysis and discussion: (i) what is the current variety offer at the agro-dealer and what drives varietal selection, (ii) what support are agro-dealers receiving to sell maize seed from suppliers, (iii) how do farmers decide which seed to buy and what is the agro-dealer influence, and (iv) how do seed companies interact with agro-dealers and farmers.

## Background

### Maize seed in Kenya

Most maize seed in Kenya comes from the formal sector. Kenya has been one of the early success stories of breeding programs, with the launch of several widely adopted hybrids in the 1960s and 1970s (Das et al., 2019; Gerhart, 1975), leading to 80% coverage of improved varieties (Das et al., 2019; Smale and Olwande, 2014; Walker and Alwang, 2015). Currently, the national variety list includes 363 maize varieties, of which over half was released in the last 10 years (KEPHIS, 2019). There has been an increase of seed companies operating in Kenya in the last 10 years, but the market continues to be dominated by Kenya Seed Company, a parastatal that holds a market share of 70–80% (Christinck et al., 2018). The volume of certified seed produced has increased from 27,078 tons in 2008 to 54,555 tons in 2017 according to Kenya Plant Health Inspectorate Service (KEPHIS). However, while seed supplies are growing in number, Kenya still imported over 7000 tons of seed in 2017 according to KEPHIS, mainly from Zambia.

Most seed companies reach farmers in Kenya through agro-dealerships (Erenstein and Kassie, 2018). Larger agro-dealers buy seed directly from the seed companies and, in addition to selling directly to farmers, resell seed to smaller, nearby agro-dealers, often called agrovets (AGRA, 2017; Christinck et al., 2018). With over 10,000 agro-dealers in the country (Bayesian Consulting Group Limited, 2016), Kenya is, together with Malawi, one of the countries in Africa with the most extensive agro-dealer network (Burke, 2016). Cultivating New Frontiers in Agriculture and the Alliance for a Green Revolution in Africa have supported the growth and development of agro-dealers, often with a focus on training to build basic business administration and marketing capacities (Makinde and Muhhuku, 2017; Odame and Muange, 2011).

However, varietal turnover has been low and the average variety age in Kenya has been consistently above 10 years in the last measurements: 17.3 years in 2010 (Smale and Olwande, 2014) and 13 years in 2013 (Abate et al., 2017). A key example of this is the case of H614. This variety was released by the Kenya Seed Company in 1986 and over the last 30 years has been one of the most popular varieties in the country (Hassan et al., 1998; Smale and Olwande, 2014; Spielman and Smale, 2017). Such dominance by a single variety is exceptional, especially given the high levels of investment that have been made in breeding and seed production over recent decades resulting in varieties with objectively higher potential yields and improved pest and disease resistance. This raises critical questions that have yet to be explored in the seed systems discourse: to what extent are farmers interested and willing to explore new seed options and how well informed are farmers about the benefits and risks associated with the various seed options available.

### The agro-dealer as entry-point for input value chain development

Although agro-dealers have been identified as key for achieving the goals of the green revolution in Kenya (Odame and Muange, 2011), reliable evidence about seed and input distribution networks remains scarce. Various studies have discussed agro-dealers’ role in subsidy programs (Jayne et al., 2018; Poulton and Macartney, 2012). But these studies focused much more on the impact of the subsidy programs on farmers, while agro-dealers were seen as a mere vehicle for implementation. However, agro-dealers in Eastern Africa are critical for seed companies to achieve scale and efficiency in seed distribution. Given low population densities and poor infrastructure in rural areas, the direct sales by seed companies in their own shops are limited (Erenstein and Kassie, 2018; Langyintuo et al., 2010; Odame and Muange, 2011). In addition to providing inputs at competitive prices for farmers, some agro-dealers provide technical assistance, such as advice on input use and production practices, and offer short-term credit for input purchases. Agro-dealers operate in a highly competitive market space and any expectations about their engagement with poor farmers must be checked by their needs to earn profits and minimize risks. These different roles and priorities can result in tensions in seed value chains, described in Table 1.

**Table 1.** Links among actors in formal maize seed systems.

	Seed producing businesses	Agro-dealers	Seed consumers
Expected role in the chain	Multiply seed Maintain seed quality Market seed	Sell seed Provide technical assistance to farmers on seed selection	Purchase appropriate seeds based on economic and agroclimatic conditions
Business relations	Agro-dealers Public agencies, NGOs, and relief programs	Upstream: Seed-producing businesses and wholesalers Downstream: Seed consumers	Agro-dealers Direct distribution from public agencies, NGOs, and relief programs
Possible tensions in relations	Weak and unreliable network for seed distribution Market distortions (e.g. fake seed, free seed provided to farmers) Delayed payments of agro-dealers	Limited incentives and capacities to market improved seed to farmers Unreliable seed supply at peak season Lack of credit for seed sales Market distortions (e.g. fake seed, free seed provided to farmers)	Limited knowledge about the benefits of improved seed Limited capacity to purchase seed and related inputs Strong loyalty to specific varieties

While seed companies rely on agro-dealers for the bulk of their seed distribution to smallholder maize farmers, it is not their only distribution pathway. Large volume sales to local or national governments, non-governmental organizations (NGOs), or relief programs (often for free seed distribution) have been common practice in Kenya (Christinck et al., 2018). While seed companies might prefer these bulk sales as they reduce marketing costs, it also reduces their willingness to invest in building their own retail networks and leads to crowding out commercial value chains (Smale et al., 2015). Also agro-dealers face several challenges. External challenges listed by Odame and Muange (2011) were a weak regulatory framework, lack of working capital, high input prices further away from supply centers, low and erratic agricultural input demand, and inadequate supply of inputs at the peak planting season. Adesina et al. (2014) touch on some of the more internal problems agro-dealers have such as limited knowledge of inputs and weak business management skills. These challenges can restrict them to act as reliable partners for seed companies.

## Material and methods

### Data collection

Data were collected in Kenya at the start of the main maize growing season (March–June 2019). Three complementary methods were used to understand agro-dealers and their interactions with seed companies and farmers: structured surveys were carried out with agro-dealer attendants (i.e. the store owner, manager, or employee), intercept interviews were implemented with farmers who visited agro-dealerships to purchase maize, and in-depth interviews were carried out with seed company owners or managers. Important to note is that the majority of maize seed in Kenya is purchased within a short period, roughly 2–3 weeks, following the first rains, which mark the start of the planting season. We adjusted our data collection to weather patterns and carried out surveys in locations where rain had started to fall. The interviews with seed companies were not restricted to this time frame.

### Agro-dealer survey

**Figure 1.**
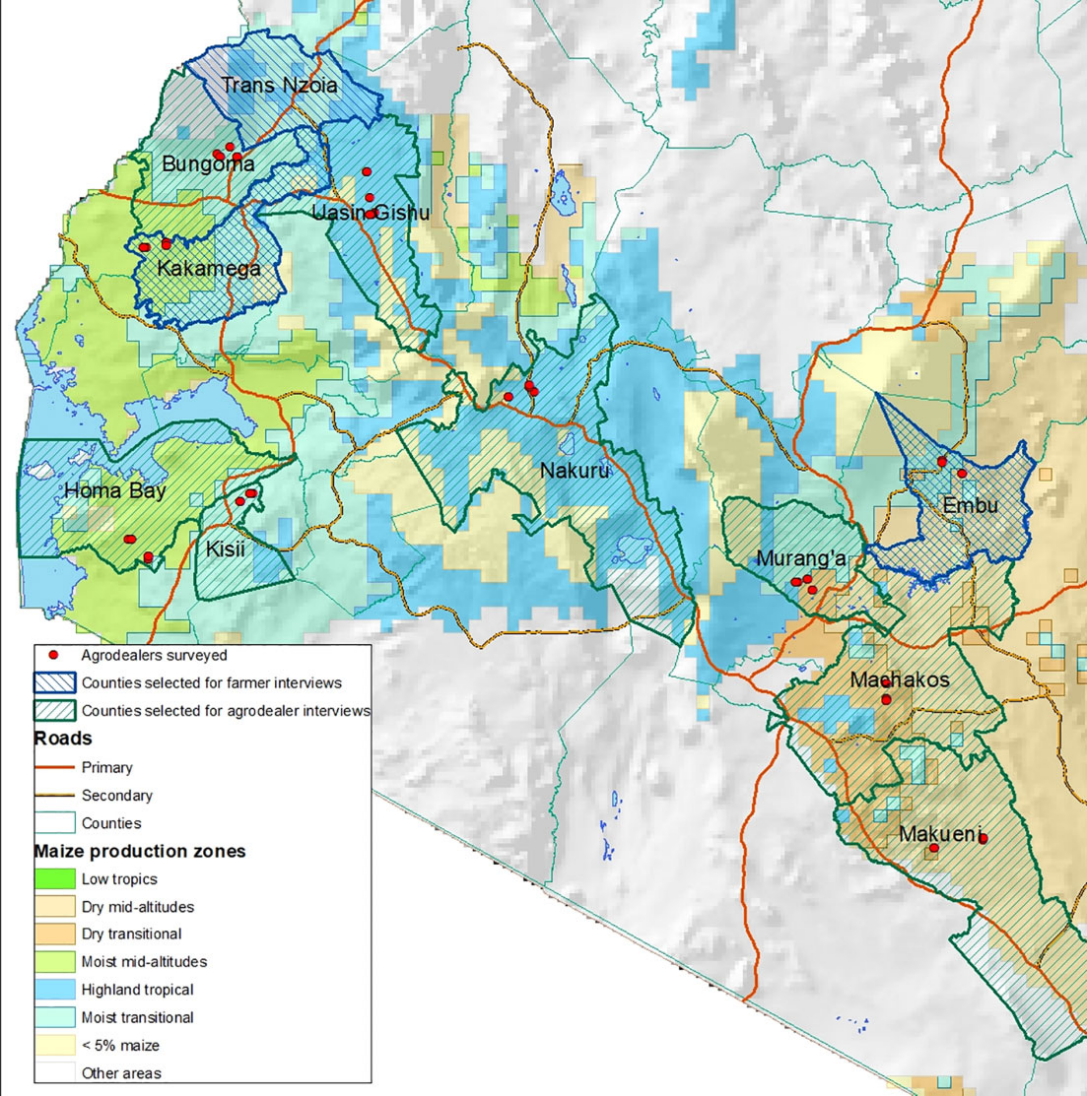
Locations of agro-dealer surveys and farmer intercept interviews.

A structured survey was carried out with 80 agro-dealers where sample design considered potential differences among agro-dealers in terms of the agro-ecological zone in which they were located and their proximity to urban centers. Two counties were randomly selected in each of the following agro-ecological zones: highlands, wet upper mid-altitude, wet lower mid-altitude, dry mid-altitude, and dry transitional. In those 10 counties, 1 sub-county was randomly selected and in that sub-county 4 agro-dealers in the sub-county main center (referred to as urban center) and 4 agro-dealers in rural centers were randomly selected from a centralized agro-dealer database of Kenya. This database was verified and adjusted where necessary by local government officials at the sub-county level. Figure 1 shows the selected counties and locations of the agro-dealers.

The agro-dealer survey covered five topics: (i) current maize seed varieties in store, (ii) selection of new maize seed varieties, (iii) promotion of new maize seed varieties, (iv) engagement with seed suppliers and customers, and (v) opportunities and challenges of the agro-dealer to increase maize seed sales. Interviews lasted roughly 60 min and information was recorded with tablets by experienced enumerators. Interviewees were screened based on their knowledge on maize sales and years of experience. Interviews were carried out with the individual who was most knowledgeable about maize seed sales and day-to-day transactions. He or she was often the store owner but the person could as well be a store manager or employee.

### Farmer intercept interviews

Intercept interviews were carried out with farmers exiting selected agro-dealers who had purchased maize seed for themselves or someone in their family and were involved in maize farming. Respondents were invited to participate when coming out of the agro-dealer in a 10–15-min survey and received a small incentive for their participation. Each farmer coming out of the agro-dealer with at least one bag of maize seed was invited to participate in the survey. Important to note here is that we did not engage with farmers that left the store without maize seed. It might have been the case that a farmers’ preferred variety was not available and that he or she would have left the store without purchase.

Three counties were selected based on their agro-ecological zone and the high prevalence of maize farming: Embu (dry mid-altitude and transitional), Kakamega (moist mid-altitude and transitional), and Trans-Nzoia (highlands), also indicated on Figure 1. In each county, the main county center and a rural location were selected, and depending on foot traffic per agro-dealer, one to three agro-dealers were selected in each location to recruit farmers. Locations of the intercept interviews were selected independent from the agro-dealer surveys. The intercept interview covered four topics: (i) maize seed purchased, (ii) factors influencing the seed purchase decision, (iii) information sources regarding maize seed, and (iv) reasons for selection of the agro-dealer.

### Seed company interviews

Semi-structured interviews were carried at eight seed companies with company owners or managers in Kenya in 2019. This is part of a larger data collection effort focusing on seed companies and other stakeholders in the maize seed sector in East Africa. These interviews lasted on average 2–3 h, covering a wide range of topics including current seed production, varietal replacement, seed distribution and marketing, production costs, and overall challenges and barriers for growth. For this discussion, only the section on seed distribution and marketing will be presented.

### Sample description

Table 2 provides basic information on the selected agro-dealer stores and the store attendants. The selected agro-dealers had, on average, 8 years in business and most stores were owned by a sole proprietor. A small share of the sample was comprised of direct agents of seed companies (Kenya Seed Company in most cases) and only 10% belonged to an agro-dealer association. For approximately half of the stores, maize seed ranked among the top three revenue generators and it was the most important revenue generator for 10%. Over 40% of the interviewees was female and the average age was 40. The interviewees had a high education level with only 10% being lower than a secondary degree and over 50% attending college or university. Almost 70% of all interviewees were owners and approximately 20% stated they were store managers; 80% received training on agriculture.

**Table 2.** Characteristics of the agro-dealers participating in the survey.

	Total
*N*	80
**Business characteristics**	
Years in business (SD)	8.3 (6.7)
Type of ownership (%)	
Sole proprietor	92.5
Partnership	5.0
Cooperative	2.5
Agent of a seed company (%)	15.0
Member of an agro-dealer association (%)	10.0
Importance of maize in revenue (%)	
Most important	10.0
In top 3	51.3
Not in top 3	48.7
**Respondent characteristics**	
Age respondent (SD)	40.0 (12.3)
Female respondent (%)	43.8
Education level (%)	
Higher than secondary	57.5
Secondary to high school	32.5
Lower than secondary	10
Position (%)	
Owner	68.8
Manager	18.8
Employee	12.4
Participated in agricultural-related training (%)	80.0

Table 3 presents the characteristics of the farmer sample participating in the intercept interviews. Roughly 33% of our sample consisted of female farmers whose average age was rather high (48 years), but consistent over our three locations. Almost 60% of farmers did not obtain a secondary degree. Farmers in Trans-Nzoia had larger maize fields and a larger portion of the maize was used for commercial production. Most farmers visited the agro-dealer by public transport and travel time was on average 30 min.

**Table 3.** Characteristics of the farmers participating in the intercept interviews.

	Total	Embu	Kakamega	Trans-Nzoia
*N*	466	172	205	89
Female (%)	33.7	37.8	33.2	27.0
Age in years (SD)	48.3 (15.6)	50.0 (15.6)	46.9 (15.4)	48.3 (17.9)
Education level (%)				
Higher than secondary	17.8	25.0	10.7	20.2
Secondary to high school	23.0	26.2	20.5	22.5
Lower than secondary	59.2	48.8	68.8	57.3
Farming experience in years (SD)	18.0 (14.6)	18.2 (14.1)	17.8 (14.6)	17.8 (15.7)
Size of maize field in acres (SD)	2.0 (2.7)	1.5 (2.2)	1.4 (1.4)	4.2 (4.3)
Part of maize harvest sold (%)	38.7	43.3	26.9	57.4
Visited urban agro-dealer (%)	53.4	57.6	54.1	43.8
Means of transport (%)				
Own transport (car, motorcycle)	20.8	25.6	17.1	20.2
Public transport	66.3	57.6	70.7	73.0
By foot or bicycle	12.9	16.9	12.2	6.7
Travel time in min (SD)	30 (28)	32 (33)	27 (25)	31 (21)

The eight seed companies that were interviewed consisted mainly of companies operating on a national level, two operating on regional level and one was a multinational. Five of them focused exclusively on seed, the three others were also involved in pesticide or grain sales. The companies were producing five different maize varieties on average and maize sales contributed for five of them to more than 70% of total revenue, including two companies who only sold maize seed.

## Results

### Agro-dealer variety offer and selection

Table 4 presents the maize varieties sold in the long season of 2018 at the selected agro-dealers. In total, 59 varieties were available in the 80 agro-dealers with an average of 8 varieties on offer at any one agro-dealer. The average age from the moment of release of these 59 varieties was 14 years. Some varieties such as Duma43 and DK8031 were available across the country, transgressing different agro-ecological zones. Varieties released in the last 10 years were not that widely spread compared to the ones that have been longer in the market. SY594 can be seen as an exception as it was only released a year before data collection and already available in one out of five agro-dealers of our sample.

**Table 4.** Agro-dealer overview of maize seed varieties in store during the 2018 long season (*n* = 80).

Number of different varieties sold in total	59
Average variety release age (SD)	14.0 (7.1)
Average number of maize varieties in store (SD)	8.0 (4.3)
Top 5 accessible varieties across agro-dealers	SC Duma 43 (15 years)—70.0%
	DK 8031 (15 years)—61.3%
	H513 (23 years)—52.5%
	H614D (32 years)—43.8%
	H6213 (16 years)—40.0%
Top 5 accessible varieties across agro-dealers released in the last 10 years (2009–2018)	H6218 (9 years)—23.8%
	SY594 (1 year)—18.8%
	DK 9089 (6 years)—12.5%
	WH 507 (8 years)—11.3%
	WE1101 (5 years)—10.0%

Four out of five agro-dealers introduced at least one new variety in the last 5 years (Table 5). On average, 3.1 varieties were introduced per agro-dealer and of all varieties introduced in the last 5 years, 16.8% was discontinued. As anticipated, “response to farmer demand” was reported to be the main driver for varietal selection by agro-dealers. Decisions to stock three out of four varieties were made based on farmer requests. The impact of demonstration plots on agro-dealer uptake of varieties was minimal. Only in a few cases did agro-dealers mention near-by demonstration plots by seed companies as an influencing factor to stock a variety in their store. Two of the top five varieties that were added to the agro-dealers’ stock were released in the last 10 years (SY594 and H6218). The others were older varieties that continued to increase the market reach by penetrating new areas.

**Table 5.** Introduction of new varieties at agro-dealers in the last 5 years (*n* = 75^a^).

Share of agro-dealers that introduced at least one new variety in the last 5 years (%)	81.3
Average number of new varieties introduced in the last 5 years (SD)	3.1 (2.7)
Share of new varieties that were discontinued (%)	16.8
Reason for adding a variety (%)	
Demand from farmers	76.7
To diversify available stock	34.9
Recommended by a seed company	33.6
Expected high returns	3.6
Promising results from demonstration plots	2.6
Top 5 varieties that were added to an agro-dealers’ stock	SY594 (1 year)—18.8%
	H522 (15 years)—16.3%
	DK8031 (15 years)—13.8%
	H6218 (9 years)—13.8%
	SC Duma 43 (15y)—12.5%

^a^ Five agro-dealers that opened in the last 3 years were left out of this analysis.

### Agro-dealer interaction with seed companies

Table 6 gives an overview of seed company engagement and support to agro-dealers in the form of information, sales, and credit support. Roughly 61% of the agro-dealers received some type of information directly from seed companies, mainly regarding the production and availability of new hybrids. Direct sales support to agro-dealer sales was markedly less common than informational sharing. The most common form of supporting sales was limited to the provision of seed marketing posters. Free seed samples, in-store promotion or supporting demonstration plots did also occur but were only mentioned by a small number of agro-dealers. Approximately one out of four agro-dealers was able to purchase (part of their) seed on credit.

**Table 6.** Percentage of agro-dealers receiving support from seed companies with information, sales, and credit (*n* = 80).

Information support last year (%)	61.3
Type of information support (%)	
Notifying about new varieties	55.0
Suitability of a variety to an agro-ecological zone	21.3
Variety performance in the field	18.8
Seed spacing/seed rate	13.8
How to identify certified seed	12.5
Circulation of uncertified/fake seeds	10.0
Seed storage	8.8
Regular sales support in the last 3 years (%)	38.8
Type of support (%)	
Provision of posters	28.8
Free seed samples	10.0
In-store promotion support	10.0
Demonstration plots	8.8
Price discounts	3.8
Credit support last year (%)	27.5

### Seed selection by farmers and agro-dealer influence

**Figure 2.**
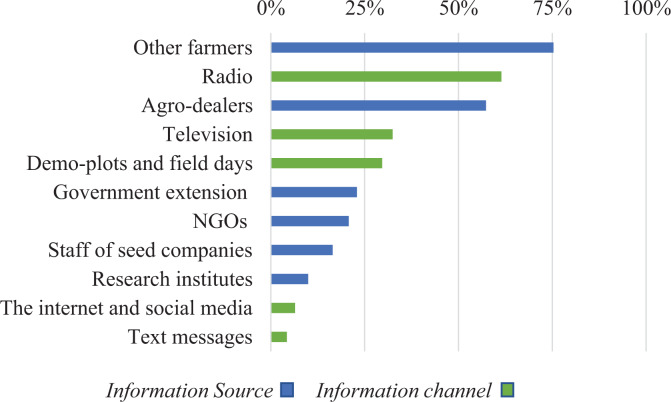
Reliable information sources and channels farmers used in the last 3 years to get information about maize seed varieties (n = 466).

Farmers used a wide range of sources and channels to inform themselves about maize seed varieties (Figure 2); the most dominant source were other farmers, mentioned by 75% of the farmers. Almost 6 out of 10 farmers used agro-dealers as an information source about seed varieties; extension officers, NGOs, research centers, and staff from seed companies did not play a big role. In terms of information channels, radio was the most influential information channel reaching approximately 60% of the farmers, followed by TV and demonstration plots. More recent methods such as text messaging or Internet and social media displayed only minimal reach to farmers with approximately 5% usage.

Although most Kenyan farmers buy their maize seed at the agro-dealer, engagement in the store at the moment of purchase tended to be limited (Table 7), suggesting that farmers generally take their decisions on seed before entering the store. Only 1 out of 10 farmers asked for information regarding their seed purchase. Even attention to the current seed offer in store was minimal. Three out of four farmers paid no attention to the available seed varieties at the agro-dealer; only 10% had a closer look at the available products.

**Table 7.** Farmer interaction at agro-dealer.

	Total	Embu	Kakamega	Trans- Nzoia
*N*	466	172	205	89
Asked for information about the seed from agro-dealers (%)				
Yes	12.7	11.6	18.0	2.2
No	87.3	88.4	82.0	97.8
Attention to varieties in store (%)				
I had a detailed look at each variety	1.5	1.7	1.0	2.2
I looked at the available offer	8.4	8.7	9.8	4.5
I had a quick look at what’s available but wouldn’t be able to retell what the varieties are	15.0	9.3	18.5	18.0
I did not look at other varieties than the one I bought	75.1	80.2	70.7	75.3

**Figure 3.**
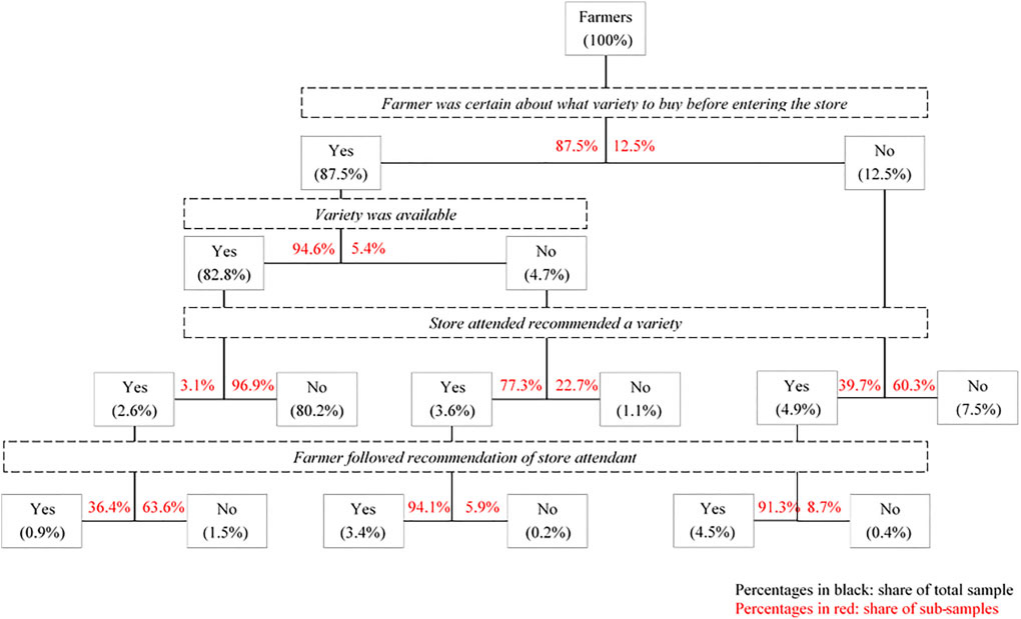
Seed purchase decision-making and agro-dealer influence (n = 466).

Figure 3 traces the seed purchase decision and the influence the agro-dealer had on that decision. Most farmers (87.5%) reported having a firm idea of which variety they wanted to purchase before they entered the store, and the vast majority of these farmers (81.7%) actually ended up actually purchasing that particular variety. For 5.4% of that group of certain buyers, that variety was not available, but they purchased a different variety in the same store.^1^ Agro-dealers barely tried to influence farmers that found their preferred variety in store.

For the 5.4% of farmers who switched seed (i.e. did not purchase the seed which they had planned to purchase) or were uncertain of which seed to purchase before entering the agro-dealership (12.5%), the influence of the agro-dealer was significant. For the group that did not find his variety in store but ended up purchasing a different variety, the agro-dealer gave advice to three-quarters of the farmers and all farmers but one followed this advice. Four out of 10 farmers that were not sure of what to buy received advice from the agro-dealer, and also here success rates were high. In total, roughly 11% of farmers received advice about varieties from agro-dealers and 9% followed that advice.

In total, 22.3% of the farmers purchased a variety for the 2019 planting season that was different from the one they bought last year (Table 8). This was highest in the rural zone of Kakamega (41.5%) where the season was already in a later stage and some of the popular varieties were no longer available. Remarkable is that roughly 60% of our participating sample switched varieties in the last 3 years and up to roughly 80% switched varieties in the last 5 years. Only a small group of farmers continued with the variety they had been growing for over 5 years and the select group had grown the same variety for 10 years or longer.

**Table 8.** Years growing the same variety.

	Total	Embu	Kakamega	Trans- Nzoia
*N*	466	172	205	89
Years growing the same variety (%)				
Bought a different variety than last year	22.3	15.7	30.7^a^	15.7
Growing the same variety for 1–3 years	38.2	33.7	41.0	40.4
Growing the same variety for 4–5 years	18.7	20.9	14.6	23.6
Growing the same variety for 6–10 years	14.4	19.8	10.7	12.4
Growing the same variety for more than 10 years	6.4	9.9	2.9	7.9

^a^ The higher % of buying a different variety was mainly due to the local center selected in Kakamega (41.5%) where already several varieties were sold out and farmer choice was limited. In the urban zone, purchase of a different variety was 21.6%.

Looking closer at farmers who purchased a different variety than last year, the results highlight the potential of the agro-dealer in switching varieties (Table 9). Although their peers were the main influencers to switch variety, the agro-dealer was the second most important trigger. Several other external information sources were much less influential on farmer decision-making. Information through radio or TV as well as extension officers or seed company demo-plots all had a minimal reported effect on switching to a new seed variety.

**Table 9.** Triggers for changing a variety.

	Triggers for buying a new variety
*N*	104
Performed well on my neighbor’s farm	29%
Recommended at the agro-dealer	28%
Recommended by the fellow farmer or relative	14%
Varietal characteristics	12%
Did not find the desired variety	4%
Learnt about the seed on radio/TV	3%
Picked the variety to see how it performs	3%
Recommended by the extension officer	3%
Saw it at a demonstration plot/ field day	2%
Recommended by the seed company	2%
Low price	1%

### Seed company interactions with agro-dealers and farmers

The most important channel for seed companies to reach farmers with their products was through agro-dealers, followed by (local) government purchases and direct sales. The seed companies worked directly with local agro-dealers, with regional distributors or with both. However, marketing efforts of seed companies were mainly targeted toward end users, that is, farmers. Most investments went into creating awareness about varieties through working with lead farmers, demonstration plots, field days, and radio. Besides developing posters or pamphlets and free seed samples, there were few activities to capitalize on their distribution networks to promote new hybrids.

Several companies perceived seed distribution as one of the key challenges for business growth. One seed company owner described agro-dealers as “*a necessary* evil”: agro-dealers were needed to move seed quickly and cheaply to farmers, but they were prone to abuse their unique position in the chain. Because maize seed sales are season bound and maize is a perishable product, seed companies must sell their complete maize stock in a very short time window. Due to low cash reserve, many of the agro-dealers are not able to pay stock upon delivery and wait until after the sales season. However, the most frequent complaint by companies was long delays in payment or even nonpayment by agro-dealers for delivered stock. Interestingly for one of the seed companies, seed distribution was not reported as a major bottleneck. This company began as a local distributor for imported vegetable seeds, pesticides, and fertilizer and only recently engaged in its own seed production. As the company already had an established distribution network and experienced in house sales team, it was relatively initiative to employ its preexisting network for maize seed sales.

## Discussion

This study presented a snapshot of interactions in the maize seed value chain in Kenya and characterized the decisions made by agro-dealers, farmers, and seed companies related to maize seed, to gain a better understanding intervention options for encouraging the purchase of new, stress-tolerant hybrids. Starting from the agro-dealer perspective, results showed that their decision-making about varieties was mainly driven by farmers, that is, demand driven. From a short-term business point of view, this approach makes sense: cash strapped agro-dealers have limited incentives to invest in products which may be harder to sell and result in lower overall seed sales. Sales support from seed companies was also limited, besides the provision of posters. The business case for agro-dealers to invest scarce time and resources to build demand among smallholders has yet to be made.

Investments that are currently being made in seed systems, development of stress-tolerant varieties and advances in technology, will be in vain if farmers as well as agro-dealers “stick with the old seed.” Simtowe et al. (2019) summarized the factors that influence adoption including farmer-oriented aspects such as risk and uncertainty, farm size, household preferences, or intra-household decision-making; as well as variety traits, information exposure, and access to seed. However, the issue of market competition has not been discussed. With an average of eight varieties per store, a long-standing tradition of buying hybrid seed and strong local as well as international players, the adoption discussion does not only depend on the farmer or the intrinsic product characteristics. It depends on a products’ ability to penetrate a saturated market that is almost entirely based on experience attributes such as germination rate, yield potential, and stress resistance. Farmers in our sample were not averse to change. Less than 20% of the farmers have been growing the same variety for longer than 5 years. This contradicts the perception that smallholder farmers absolutely want to stick to what they know and are averse to change (Das et al., 2019). So there are opportunities to influence farmer choices and create demand for new varieties.

Seed companies made use of agro-dealers to distribute their products but much less for the promotion of these products. In many cases, input producers have bypassed retailers altogether, to engage directly with farmers. Their investments went mainly into demonstration plots, field days, and radio commercials, all focused toward the end-user. This method is traditionally known as “pull marketing,” creating demand by convincing the end user to look for the product (Ailawadi et al., 2009; Kopp and Greyser, 1987; Olver and Farris, 1989). “Push marketing” on the other hand is a strategy to “push” products to end users and make sure that products are standing out at the point of purchase. This methodology is often used to create initial product exposure but requires a strong sales team, attracting point of sales material as well as negotiating power with distributors (Ailawadi et al., 2009). These strategies have been discussed for over 30 years in the retail and marketing research but to our knowledge have not been explored in seed systems. While sources above highlight the need to have push and pull marketing in combination, only pull marketing appears to be present in maize seed markets.

Also from a farmers’ perspective, the retail environment was not considered when making the seed choice, with minimal attention being focused on the available seed offer or effort being made to obtain information on seed performance. This raises some important questions: Would future interventions be better off supporting directly marketing by seed companies, with a passive role for agro-dealers in distribution? Or, should interventions seed to build stronger links between seed companies and agro-dealers for push marketing of new hybrids to smallholders? In the absence of effective extension programs (Tata and McNamara, 2018), smallholders in developing regions have scarce information when taking critical decisions on agricultural input use, including veterinary supplies, fertilizers, agrochemicals, and seeds. Also in our sample, agro-dealers appeared to be one of the few external information sources with sufficient reach to farmers. Secondly, agro-dealers were not giving advice about seed selection to farmers that often, but were quite successful in changing behavior when they did. Better use of the retail environment was also the goal of the recent SEEDS project focusing on seed marketing at agro-dealers in Mozambique (NCBA CLUSA, 2017). Results have been promising with an increase in sales as well as farmer demand. Also, a recent McKinsey report on Africa’s agricultural market highlights the pivotal role of the agro-dealer in increasing productivity (Goedde et al., 2019), stressing increased use of key elements of push marketing such as better business relationships, more flexible payments, and improved services to agro-dealers.

All of this suggests that agro-dealers should not be overlooked to promote seed. When farmers enter the agro-dealer store, they become customers with their own motives, needs, and preexisting ideas on the best seed, all of which can be potentially influenced in store (Inman et al., 2009). Taking advantage of this will require stronger collaboration between retailers and seed businesses and deeper insights into which marketing approaches work best in a different context. Interventions in the retail environment, such as targeted marketing materials, provision of in-store decision support, price incentives, and promotions both to retail partners as to end users need to be further investigated and evaluated to improve the competitiveness of new products in input markets.

## Conclusion

New maize seed varieties face an uphill march to gain ground against the market leaders. There have been lots of investment in seed systems, however, the bulk of these investments are concentrated on seed producers. While it is important to have a steady supply of quality stress-tolerant seed, it is not enough to change varietal turnover. More changes are needed in the regulatory environment and seed producers might still have unmet needs. But there is also the distribution side that needs to be adequately addressed. And big gains in expanding the formal seed systems could be made here. Research shows the potential as well as the long road ahead for the seed retail sector to play a more substantial role. Seed companies need to do more to engage with their distribution networks and leverage their important role in variety uptake. Innovations are needed in how they engage with retailers and pursue more active marketing approaches to farmers or improve their business to business approaches.

## Note

1. As mentioned before, we did not engage with farmers who left the store without maize seed. It might have been the case that a farmers’ preferred variety was not available and that he or she would have left the store without a purchase.
